# Modulation of Tongue Pressure According to Liquid Flow Properties in Healthy Swallowing

**DOI:** 10.1044/2018_JSLHR-S-18-0229

**Published:** 2019-01-25

**Authors:** Catriona M. Steele, Melanie Peladeau-Pigeon, Carly A. E. Barbon, Brittany T. Guida, Melanie S. Tapson, Teresa J. Valenzano, Ashley A. Waito, Talia S. Wolkin, Ben Hanson, Jane Jun-Xin Ong, Lisa M. Duizer

**Affiliations:** a Toronto Rehabilitation Institute—University Health Network, Swallowing Rehabilitation Research Laboratory, Ontario, Canada; b Speech-Language Pathology, Rehabilitation Sciences Institute, University of Toronto, Ontario, Canada; c University College London Mechanical Engineering, United Kingdom; d Department of Food Science, University of Guelph, Ontario, Canada

## Abstract

**Purpose:**

During swallowing, the tongue generates the primary propulsive forces that transport material through the oral cavity toward the pharynx. Previous literature suggests that higher tongue pressure amplitudes are generated for extremely thick liquids compared with thin liquids. The purpose of this study was to collect detailed information about the modulation of tongue pressure amplitude and timing across the range from thin to moderately thick liquids.

**Method:**

Tongue pressure patterns were measured in 38 healthy adults (aged under 60 years) during swallowing with 4 levels of progressively thicker liquid consistency (International Dysphagia Diet Standardisation Initiative, Levels 0 = *thin*, 1 = *slightly thick*, 2 = *mildly thick*, and 3 = *moderately thick*). Stimuli with matching gravity flow (measured using the International Dysphagia Diet Standardisation Initiative Flow Test; Cichero et al., 2017; Hanson, 2016) were prepared both with/without barium (20% weight per volume concentration) and thickened with starch and xanthan gum thickeners.

**Results:**

After controlling for variations in sip volume, thicker liquids were found to elicit significantly higher amplitudes of peak tongue pressure and a pattern of higher (i.e., steeper) pressure rise and decay slopes (change in pressure per unit time). Explorations across stimuli with similar flow but prepared with different thickeners and with/without barium revealed very few differences in tongue pressure, with the exception of significantly higher pressure amplitudes and rise slopes for nonbarium, starch-thickened slightly and mildly thick liquids.

**Conclusions:**

There was no evidence that the addition of barium led to systematic differences in tongue pressure parameters across liquids with closely matched gravity flow. Additionally, no significant differences in tongue pressure parameters were found across thickening agents.

**Supplemental Material:**

https://doi.org/10.23641/asha.7616537

For individuals who experience airway invasion when swallowing thin liquids (i.e., penetration or aspiration), the most common clinical intervention is to recommend thickened liquids (Garcia, Chambers, & Molander, 2005; Robbins et al., 2002). The premise behind using thickened liquids is that increased viscosity (i.e., resistance to flow) makes these liquids easier to control and that their slower flow provides additional time in which to achieve laryngeal vestibule closure, thereby reducing the risk of penetration–aspiration. However, recent evidence also suggests that there is increased risk of postswallow residue in the pharynx with very thick liquids (Hind et al., 2012). Although diet texture modification has become a cornerstone of dysphagia management, evidence to guide clinicians in choosing appropriate consistencies for clinical management is lacking (Robbins et al., 2002; Steele et al., 2015). A recent systematic review published by the International Dysphagia Diet Standardisation Initiative (IDDSI, http://www.iddsi.org; Steele et al., 2015) shows a gap in knowledge regarding the impact of viscosity and other flow properties on swallowing. Although differences in swallowing physiology are seen in comparisons between thin and extremely thick liquids, it remains unknown whether flow-related modulation of swallowing behaviors occurs for comparisons that fall between these extremes and span smaller differences along the flow continuum (Chi-Fishman & Sonies, 2002; Steele, Bailey, & Molfenter, 2010; Steele, Molfenter, Peladeau-Pigeon, Polacco, & Yee, 2014). The IDDSI review concluded that a critical need exists for new studies that explore the physiological and functional consequences of thickening liquids in both healthy and disordered populations (Steele et al., 2015).

Clinically, it is important to be able to group dietary liquids into those with similar versus different physiological flow behavior. This enables the matching of assessment materials to dietary liquids and guides decisions about the inclusion or exclusion of specific liquids when planning diets for patients with dysphagia. Recently, the IDDSI introduced a new taxonomy for classifying the thickness of liquids used in dysphagia management according to gravity flow (Cichero et al., 2017; Hanson, 2016). Measures of gravity flow are considered suitable as a model for representing fluid flow through the pharynx (in a person sitting upright), given their ability to capture information related both to shear and to extensional viscosity (Hanson, 2016; Waqas, Wiklund, Altskar, Ekberg, & Stading, 2017). The IDDSI has adopted a simple test (the “IDDSI Flow Test”; Cichero et al., 2017; Hanson, 2016), which can be used to classify liquids based on the height of the residual fluid column after allowing 10 cc of material to flow for 10 s through a standardized 10-ml slip tip syringe: Level 0 thin liquids leave 0–1 ml of residual fluid, Level 1 slightly thick liquids leave 1–4 ml, Level 2 mildly thick liquids leave 4–8 ml, and Level 3 moderately thick liquids leave 8–10 ml. Level 4 extremely thick liquids show no flow through the syringe in 10 s; additional supplementary spoon tilt and fork drip tests are recommended by the IDDSI to confirm the classification of very thick liquids as Level 3 versus Level 4 (Cichero et al., 2017; Hanson, 2016).

The purpose of the current article is to describe the patterns of tongue pressure that are seen in healthy swallowing with liquids across the flow continuum from thin to moderately thick consistency, as defined by the IDDSI framework (Cichero et al., 2017). Specifically, we wanted to understand modulations in the amplitude and pressure/time slopes of tongue pressure that occur as a function of liquid thickness. This is the first study in a planned series to establish reference data from healthy adults regarding swallowing across the IDDSI liquid levels: Level 0 = *thin* (TN0), Level 1 = *slightly thick* (ST1), Level 2 = *mildly thick* (MT2), and Level 3 = *moderately thick* (MO3). Level 4 = *extremely thick liquids* are omitted from the current investigation because their thickness cannot be quantified using the IDDSI Flow Test (Cichero et al., 2017; Hanson, 2016). Our main hypothesis was that we would see higher tongue pressure amplitudes and higher (i.e., steeper) slopes of pressure rise and decay for thicker liquids compared with thinner liquids across the IDDSI continuum.

The main hypotheses for this study are informed by a previous study (Steele, Bailey, & Molfenter, 2010) in which healthy young adults were observed to modulate tongue–palate pressure between swallows of water and a starch-thickened apple juice with a viscosity of 497 mPa·s at 50 reciprocal seconds. Specifically, higher pressure amplitudes and higher (i.e., steeper, more rapid) slopes of pressure rise and decay were seen with the thicker liquid. However, other factors were recognized to have potentially relevant, confounding influences; these include variations in sip volume, participant age and sex, the use of starch versus xanthan gum thickeners (Vilardell, Rofes, Arreola, Speyer, & Clave, 2016), and the use of barium. As a secondary goal, we wanted to explore whether differences in tongue pressure would be seen across liquids with closely matched gravity flow properties (operationally defined as a group of liquids with gravity flow results spanning not more than a 1-ml range), but with differences in other flow and material characteristics related to the use of barium and of different thickening agents (starch vs. xanthan gum).

Sip volume is commonly controlled, both in swallowing assessment protocols and in research. There are numerous previous reports suggesting that variations in sip volume influence swallowing behaviors (Barikroo, Carnaby, & Crary, 2015; Dantas & Dodds, 1990; Dantas et al., 1990; Dodds et al., 1988; Ertekin et al., 1997; Gumbley, Huckabee, Doeltgen, Witte, & Moran, 2008; Hiss, Treole, & Stuart, 2001; Kahrilas, Lin, Chen, & Logemann, 1996; Molfenter & Steele, 2014; Nagy, Molfenter, Peladeau-Pigeon, Stokely, & Steele, 2014). In this study, we wanted to control for sip volume variations but allow participants to take natural sip sizes. We expected that smaller sip volumes would be seen with thicker liquids compared with thin liquid and with barium compared with nonbarium stimuli (Steele & van Lieshout, 2005), and we expected that variations in sip volume might influence tongue pressure measures in this study.

In radiographic or endoscopic studies of swallowing, it is possible to identify situations in which more than one swallow is performed for a single bolus. This is not possible without imaging and was something that we recognized we would not be able to control in this study. For this reason, we decided to perform all measures on a single swallow for each bolus.

With respect to possible age- or sex-related differences in the magnitudes of tongue pressure, several studies in the literature suggest that older adults and women have lower tongue strength as measured by maximum isometric pressure tasks (Adams, Mathisen, Baines, Lazarus, & Callister, 2013; Fei et al., 2013; Vanderwegen, Guns, Van Nuffelen, Elen, & De Bodt, 2013) but that these differences are not seen in the submaximal pressure context in swallowing (Steele, Bailey, & Molfenter, 2010; Steele, Bailey, Molfenter, Yeates, & Grace-Martin, 2010; Youmans & Stierwalt, 2006). Consequently, we hypothesized that there would be no differences between male and female participants and no effect of age within the healthy sample (aged under 60 years) recruited for this study.

Recent studies show that it takes fewer grams of xanthan gum–based thickener than starch thickener to achieve similar target viscosities (Leonard, White, McKenzie, & Belafsky, 2014; Vilardell et al., 2016). Gum- versus starch-thickened liquids differ in terms of perceived slipperiness (Ong, Steele, & Duizer, 2018) and perceived ease of swallowing (Nystrom, Waqas, Bulow, Ekberg, & Stading, 2015), as well as in rheological characteristics such as density, yield stress, and extensional viscosity (Garcia, Chambers, Matta, & Clark, 2007; Mackley et al., 2013; O'Leary, Hanson, & Smith, 2010). Yield stress is a characteristic in which a threshold of applied stress must be surpassed before flow can begin (Cichero, Jackson, Halley, & Murdoch, 2000). Extensional viscosity refers to the ability of a liquid to stretch into a strand or filament without breaking (Hanson, 2016; Waqas et al., 2017). Furthermore, starch- and xanthan gum–thickened liquids may behave quite differently when barium is added, depending on the ingredients in the barium product (Ekberg et al., 2009; Popa Nita, Murith, Chisholm, & Engmann, 2013); in particular, studies have reported that further thickening occurs when barium is added to starch-thickened liquids (Steele, Molfenter, Peladeau-Pigeon, & Stokely, 2013).

In clinical videofluoroscopic swallowing studies, barium is commonly used as a radio-opaque contrast medium. The validity of the videofluoroscopy rests heavily on the assumption that swallowing behaviors seen while swallowing barium are representative of swallowing behaviors that occur with normal, nonbarium liquids and foods outside the assessment context. However, the addition of barium sulfate powder to a thin liquid alters taste (Ekberg et al., 2009; Nagy, Steele, & Pelletier, 2014a) and is also known to alter rheological characteristics such as density, shear thinning, and extensional viscosity (Cichero, Burey, Nicholson, Halley, & Tobin, 2011; Cichero et al., 2000; Ekberg et al., 2009; Frazier et al., 2016; Steele, van Lieshout, & Goff, 2003; Stuart & Motz, 2009). Furthermore, commercially available barium sulfate products commonly include other ingredients to aid suspension, limit foaming, and achieve desired degrees of mucosal coating. The concentrations of these additional ingredients in the barium product are usually not disclosed on manufacturer labels, but these ingredients may impact the flow characteristics of the resulting suspension. High concentrations of barium (often referred to as high-density barium) are likely to have a greater impact on both viscosity and density. It has been argued that the ideal thin liquid barium contrast medium for oropharyngeal imaging is a low concentration product that is more likely to flow in a manner similar to water (Fink & Ross, 2009) and less likely to leave a coating on the walls of the oropharynx (Steele et al., 2013). The literature suggests that significantly longer bolus transit times are seen with a 250% weight per volume (w/v) concentration compared with a 140% w/v barium concentration (Dantas, Dodds, Massey, & Kern, 1989). More recent studies have explored differences between “thin” (i.e., 40% w/v) and more dilute “ultrathin” (i.e., ~20% w/v) barium suspensions and have reported longer durations for swallow timing measures with the higher concentration product (Steele & van Lieshout, 2005; Stokely, Molfenter, & Steele, 2014). For these reasons, we wanted to explore differences in tongue pressure behaviors across stimuli with and without low concentration barium and prepared using both starch- and xanthan gum–based thickeners.

## Method

### Sample Size and Inclusion Criteria

This article includes data for a sample of healthy adults aged under 60 years. The protocol received human subjects approval from the local institutional research ethics board. Sample size calculations were performed using Study Size 2.0.5 software (CreoStat HB, 2001-2012, V.Frolunda) based on a previous study of tongue pressure modulation (Steele et al., 2014) and suggested that 36 participants per group would be required to detect differences greater than or equal to 10 mmHg in tongue pressure amplitudes with 80% power (α = .05) and a medium effect size (Cohen's *d* ≥ 0.5; Kotrlik & Williams, 2003). We therefore decided to recruit *n* = 40 for the young participant sample involved in this study, with a view to using this sample as a reference in future studies exploring age- or dysphagia-related differences.

Participants were accepted into the study, provided that they reported no current or prior history of swallowing, motor speech, gastro‐esophageal or neurological difficulties, or extreme oral sensitivity. Individuals with radiation to the neck or a history of surgery to the speech or swallowing apparatus (other than routine tonsillectomy or adenoidectomy) were excluded. Individuals with Type 1 diabetes were excluded due to the requirement to swallow stimuli containing thickeners that may carry a significant carbohydrate load. Similarly, individuals with known allergies to cornstarch, potato starch, xanthan gum, or milk products were excluded due to the known or possible inclusion of these food items in the commercial thickening agents used for the study, as specified by the manufacturer on the product label. Similarly, individuals with known allergies to latex, barium, food coloring, or dental glue were excluded, due to the possibility that these items would come into contact with the oral mucosa during data collection. The protocol specified that individuals with full upper plate dentures, who were unable or unwilling to remove their dentures for the experiment, would be excluded to avoid any damage to dental prostheses related to the attaching of sensors to the palate; however, this exclusion was not required for the participants in this sample, because none of them wore dentures. Women who were pregnant were excluded due to the use of radiation in one of the study experiments (not described in this article). Medications were reviewed, and individuals who were taking antiparkinsonian or neuroleptic medications were excluded, together with individuals who reported dry mouth as a side effect of current medication use. All participants provided written informed consent prior to data collection.

### Stimuli

Four different arrays of stimuli were prepared for this study as follows:

1. “Non-barium starch” array: commercially available lemon-flavored water (Nestlé Lemon Splash; IDDSI Level 0 = *thin*) and thickened to IDDSI Levels 1 (*slightly thick*), 2 (*mildly thick*), and 3 (*moderately thick*) using a starch-based thickener (Nestlé Resource ThickenUp). Lemon-flavored water was chosen to make these stimuli more palatable than unflavored thickened water. The taste of the unthickened commercial product was rated as mild by a blinded taste panel, who judged the intensity of the sourness and sweetness to be similar to a solution of 0.02% lemon juice and 0.02% sucrose in water. This intensity of sourness falls well far below the levels reported to induce chemesthesis and impact swallowing behaviors (Nagy et al., 2014a; Nagy, Steele, & Pelletier, 2014b; Pelletier, 2007; Pelletier & Dhanaraj, 2006).
2. “Non-barium xanthan” array: the same commercially available lemon-flavored water (Nestlé Lemon Splash; IDDSI Level 0 = *thin*) thickened to IDDSI Levels 1 to 3 using a xanthan gum thickener (Nestlé Resource ThickenUp Clear).
3. “Barium starch” array: a liquid barium suspension (IDDSI Level 0 = *thin*) developed using Bracco E-Z-Paque 96% w/w barium powder, added to bottled water (Nestlé Pure Life) in a 20% w/v concentration (Fink & Ross, 2009) and thickened to IDDSI Levels 1 to 3 using the starch-based thickener (Nestlé Resource ThickenUp).
4. “Barium xanthan” array: the same 20% w/v liquid barium suspension (IDDSI Level 0 = *thin*) thickened to IDDSI Levels 1 to 3 using the xanthan gum thickener (Nestlé Resource ThickenUp Clear).

Mixing was performed according to a standard operating procedure in which weighed dry ingredients were added slowly (over 30–40 s) to water that was moving at a slow speed (i.e., 60 rpm) on a Bosch kitchen stand mixer and then left to mix at 60 rpm for an additional 2 min. For recipes involving barium, the barium was added prior to the thickening agent. In order to maximize the match of gravity flow within each IDDSI level across arrays, recipes for the thickeners were titrated to achieve flow test results that spanned not more than a 1-ml range. Gravity flow testing was conducted in triplicate at 1, 2, and 3 hr post mixing to ensure that the recipes produced liquids that remained within the targeted range for 3 hr at room temperature. These tests were performed using a 10-ml slip tip syringe (BD model 301604, barrel length from the 0- to 10-ml line = 61.5 mm) according to the instructions on the IDDSI website (http://www.iddsi.org). Details regarding viscosity and density of the test stimuli have been published elsewhere (Ong et al., 2018) but are summarized in Table 1.

**Table 1.** Flow characteristics of the stimuli used in the study.

IDDSI level	Barium	Thickener type	Thickener amount (g/100 ml)	Flow test residual fluid (ml)*	Viscosity at 50/s (mPa·s)*	Density (g/ml)
0 = TN0	Nonbarium	None	None	0.0	1	1.00
	Barium	None	None	0.0	3	1.16
1 = ST1	Nonbarium	Starch	4.15	1.9	75	1.01
	Nonbarium	Xanthan gum	0.65	1.9	48	1.00
	Barium	Starch	2.85	1.8	120	1.16
	Barium	Xanthan gum	0.40	1.7	51	1.17
2 = MT2	Nonbarium	Starch	4.77	5.0	141	1.02
	Nonbarium	Xanthan gum	1.10	5.2	128	1.02
	Barium	Starch	3.75	4.9	273	1.17
	Barium	Xanthan gum	0.90	5.3	157	1.15
3 = MO3	Nonbarium	Starch	5.85	9.4	338	1.03
	Nonbarium	Xanthan gum	2.10	9.1	290	1.02
	Barium	Starch	5.10	9.5	850	1.17
	Barium	Xanthan gum	2.20	9.6	361	1.16

*Note.* IDDSI = International Dysphagia Diet Standardisation Initiative; TN0 = thin; ST1 = slightly thick; MT2 = mildly thick; MO3 = moderately thick.

* Results represent mean values across three repeated tests per stimulus at 25 °C. Additional details regarding the testing methods and results can be found in Ong et al. (2018).

### Data Collection

The experiment involved measurement of tongue–palate pressure using the lingual manometry module of the KayPENTAX Swallowing Signals Lab. As described in previous studies (Steele et al., 2014), a splineless silicon strip housing three pressure bulbs was attached to the participant's hard palate in midline using an adhesive strip (Stomahesive, Convatec). The anterior pressure bulb was positioned on the alveolar ridge with the posterior pressure bulb located 4 cm posterior to the anterior bulb, in the vicinity of the junction between the hard and soft palate. The system was calibrated at the beginning of each session to measure tongue–palate pressures up to a ceiling of 500 mmHg, and data were registered at a sampling frequency of 250 Hz. Before administering any boluses, a tongue pressure signal quality check was performed by asking the participant to perform a single maximum effort anterior tongue–palate pressure task.

Participants were given both barium and nonbarium stimuli to swallow (14 in total) with each stimulus presented in a block of three boluses (i.e., 42 boluses in total). Participants were randomly assigned to one of four orders of stimulus presentation, which counterbalanced the order of the four arrays. The order of the IDDSI level was held constant within each array, progressing from thin to moderately thick. For each thin, slightly thick, and mildly thick bolus, participants were handed a cup containing 40 ml of fluid and were instructed to take a single comfortable, natural sized sip from the cup and swallow when they were ready. For each moderately thick bolus, participants were handed a cup containing 60 ml of fluid and a plastic teaspoon (confirmed to have a capacity of 5 ml with water) and were instructed to take a single teaspoonful of the stimulus and swallow when they were ready. Presip and postsip cup weights were used to determine sip volume for all stimuli.

### Data Processing

The KayPENTAX Swallowing Signals Lab software generates separate waveform traces for pressures measured at the anterior, posterior, and midpalate. Previous studies have shown that pressures at the midpalate are highly variable, perhaps due to variations in palatal vault height (Pitts, Stierwalt, Hageman, & LaPointe, 2017; Steele, Bailey, & Molfenter, 2010; Steele, Bailey, Molfenter, Yeates, et al., 2010); consequently, only anterior and posterior pressures were included in the analysis for this study. As a first step, before extracting amplitude and slope measures from the tongue pressure signals, we conducted a descriptive analysis to determine which sensor (anterior vs. posterior) registered the highest peak pressure for each bolus recording and to explore pressure sequencing patterns, according to which sensor registered the initial pressure onset, initial pressure peak, terminal pressure peak, and terminal pressure offset. These preliminary analyses revealed variations both with respect to which sensor registered the highest peak pressure (20% anterior, 80% posterior) and in the pattern of pressure sequencing. The initial pressure onset and the initial peak pressure were typically, but not always, seen at the anterior sensor (75% and 65% of the time, respectively). The terminal offset of pressure was most commonly seen at the posterior sensor (67% of the time). In view of the fact that patterns of peak pressure location and pressure sequencing were variable, we decided to derive parameters of tongue pressure amplitude and slope in an algorithmic fashion as follows:

- *Peak pressure amplitude* (in mmHg) was extracted based on the highest peak pressure seen across both sensors.
- *Pressure range* was calculated (in mmHg) as the difference between the lowest baseline and highest peak pressure seen across both sensors. This parameter was not used as a dependent variable, but was calculated in order to derive measures of *pressure slope* (below).
- *Pressure rise time* was calculated as the time interval (in milliseconds) between the initial pressure onset and the initial peak pressure. As with *pressure range,* this parameter was used for the calculation of *pressure rise slope* and not as a primary parameter of interest.
- *Pressure decay time* was calculated as the time interval (in milliseconds) between the terminal peak pressure and the terminal pressure offset. This parameter was used for the calculation of *pressure decay slope*.
- *Pressure rise slope* was calculated (in mmHg/s) as *pressure range* divided by *pressure rise time*.
- *Pressure decay slope* was calculated (in mmHg/s) as *pressure range* divided by *pressure decay time*.

**Figure 1.**
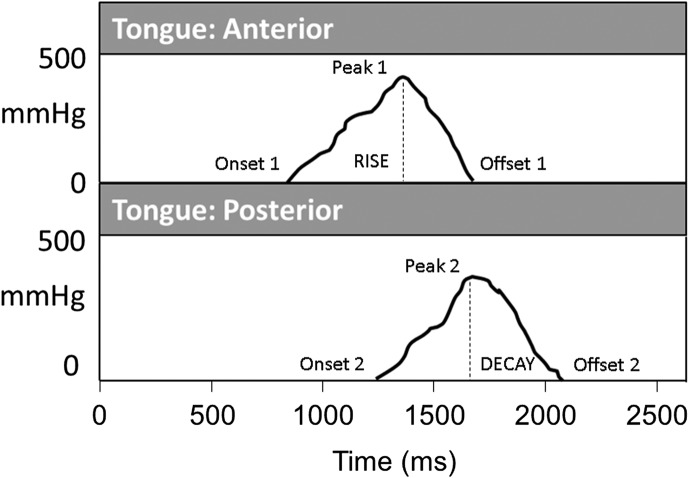
Illustration of tongue pressure waveforms and parameter derivation. In this schematic illustration, time-synchronized pressure waveforms for anterior and posterior tongue–palate pressure are displayed. For the current study, peak pressure was defined as the highest pressure amplitude obtained across both signals. The pressure rise phase was defined as the interval between the earliest pressure onset (Onset 1) and the earliest pressure peak (Peak 1). Pressure rise slope was calculated as the difference in pressure amplitude between these two events, divided by the duration of the rise phase. Pressure decay slope was derived similarly as the difference in pressure amplitude between the terminal pressure peak (Peak 2) and the terminal pressure offset (Offset 2), divided by the duration of the decay phase.

Figure 1 provides a visual illustration of the derivation of these parameters across a 2.5-s time interval.

### Analysis

Statistical analyses were performed using SPSS software (Version 24). Initial explorations of the data showed skewed distributions of residuals for all tongue pressure parameters; a log-transformation was confirmed to mitigate this concern and was applied to all of the dependent variables. As a first step, univariate mixed-model, repeated-measures analyses of variance (ANOVAs) of sip volume with a factor of the IDDSI level were conducted to determine whether sip volume needed to be incorporated as a covariate in the statistical models testing for IDDSI level effects on tongue pressure; these identified significantly smaller sip volumes for thicker consistencies (*p* < .001). Consequently, we decided to control for the impact of variations in sip volume by dividing all tongue pressure parameters by sip volume and expressing the data in *units of tongue pressure per mill* *iliter*.

Univariate mixed-model, repeated-measures ANOVAs of the tongue pressure parameters with a factor of sex were also run as a preliminary step to determine whether sex needed to be carried forward as a factor in the statistical models testing for IDDSI level effects. These explorations failed to identify any statistically significant differences between male and female participants for log peak pressure per milliliter (*p* = .28), log rise slope per milliliter (*p* = .31), or log decay slope per milliliter (*p* = .07). Similarly, Pearson product–moment correlation coefficients were explored between the tongue pressure parameters and the potential covariate of age. These revealed only weak correlations for all three tongue pressure parameters (i.e., log peak pressure per milliliter: *R* = −.25; log rise slope per milliliter: *R* = −.17; log decay slope per milliliter: *R* = −.15). On this basis, the decision not to carry sex and age forward as factors in the final statistical models was confirmed.

The main analysis approach involved linear mixed-model, repeated-measures ANOVAs within array with a fixed effect of an IDDSI level (0 to 3). Participant number was included as a random effect to control for heterogeneity across individuals. A covariate of bolus number within the array was included to control for possible order effects. A compound symmetry covariance structure was used for all ANOVA tests. Post hoc Sidak tests were used to explore pairwise comparisons, and Cohen's *d* was calculated as a measure of effect size (Kotrlik & Williams, 2003). A Bonferroni correction (i.e., α = .05/3; *p* < .017) was applied to correct for probable nonindependence between the three tongue pressure parameters of interest.

Subsequent explorations of differences in tongue pressure parameters across arrays were conducted using mixed-model, repeated-measures ANOVAs with a fixed effect of stimulus. Participant number was again used as a random effect. As with the within-array analyses, post hoc Sidak tests were used to explore pairwise comparisons, Cohen's *d* was calculated as a measure of effect size, and an alpha criterion of *p* < .017 was used.

## Results

### Participants

Forty adults (20 male, 20 female) consented to participate in the study. Mean age for the overall sample was 34 years (range: ages 21–58 years). When stratified by sex, the mean age was 35 years for the men (range: ages 21–58 years) and 33 years for the women (range: ages 25–54 years). Two male participants withdrew from the study after consent and did not complete data collection.

### Sip Volume

**Figure 2.**
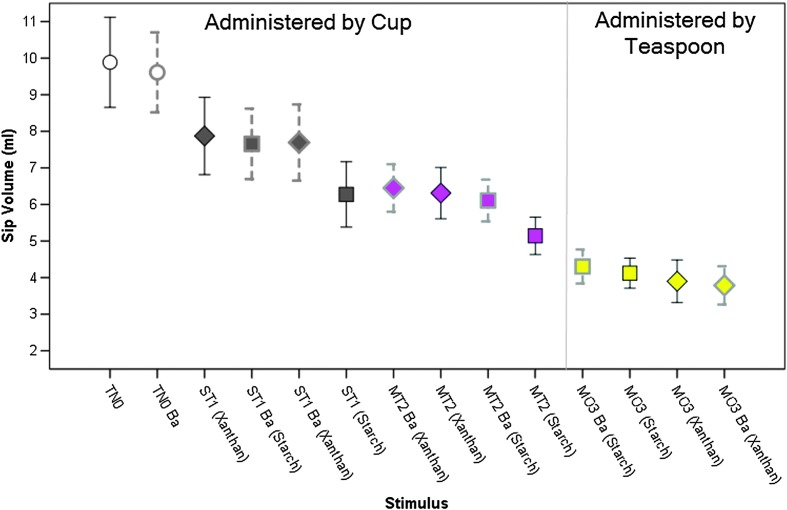
Means and 95% confidence interval boundaries for sip volume by stimulus. Significantly smaller sip volumes (*p* < .05) were seen for thicker liquids. TN0 = thin; ST1 = slightly thick; MT2 = mildly thick; MO3 = moderately thick; Ba = barium.

Figure 2 illustrates the means and 95% confidence intervals for sip volume by stimulus. The graph clearly shows that sip volumes were significantly larger for the liquids taken by cup sip (i.e., thin, slightly thick, and mildly thick) than for the moderately thick stimuli, which were taken by teaspoon, *F*(1, 1555.99) = 408.36, *p* < .001, *d* = 0.81 (large). This is probably attributable to the natural upper limit on the amount of liquid that can be loaded on a 5-ml teaspoon, given the fact that IDDSI Level 3 moderately thick liquids will flow off a utensil. In addition to this main effect of administration method, significantly smaller sip volumes (with small to large effect sizes) were also found for each incremental level of thickening within the cup sip administration method, *F*(2, 1098.99) = 161.84, *p* < .001, *d* ≥ 0.33. As described in the Method section above, these findings led to the decision to neutralize the potential confounding influences of bolus administration method and variations in sip volume on any differences in tongue pressure by expressing the data in units of tongue pressure per milliliter.

### Within Array Variations in Tongue Pressure Measures

**Figure 3.**
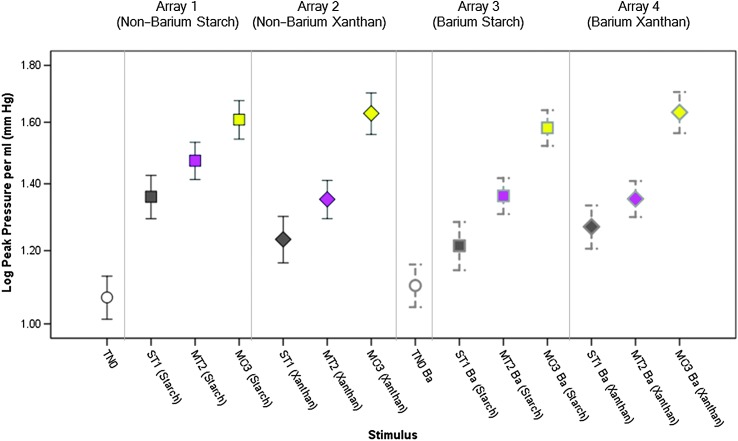
Means and 95% confidence interval boundaries for peak pressure amplitude by liquid flow level (within array). Significant increases in peak tongue pressure amplitude (*p* < .017) were seen for thicker liquids. Additional details regarding pairwise comparisons can be found in Table 2. TN0 = thin; ST1 = slightly thick; MT2 = mildly thick; MO3 = moderately thick; Ba = barium.

A table of descriptive statistics for the tongue pressure parameters of interest (both in base and log-transformed units), by stimulus, can be found in the online Supplemental Material S1. Figures 3 [Fig F4]–5 illustrate the main effects of IDDSI level on the tongue parameters of interest, by array. In general, there was a pattern of significantly higher peak pressures for thicker consistencies and patterns of higher (i.e., steeper, more rapid) pressure rise slopes and pressure decay slopes with thicker consistencies. Table 2 lists the specific comparisons that achieved statistical significance at *p* < .017. A single significant finding of an order effect was found for pressure decay slope with the xanthan gum–thickened barium stimuli. The pattern was of higher slopes for the final block of stimuli in the array (moderately thick) and occurred despite the counterbalancing of array order across participants.

**Figure 4. F4:**
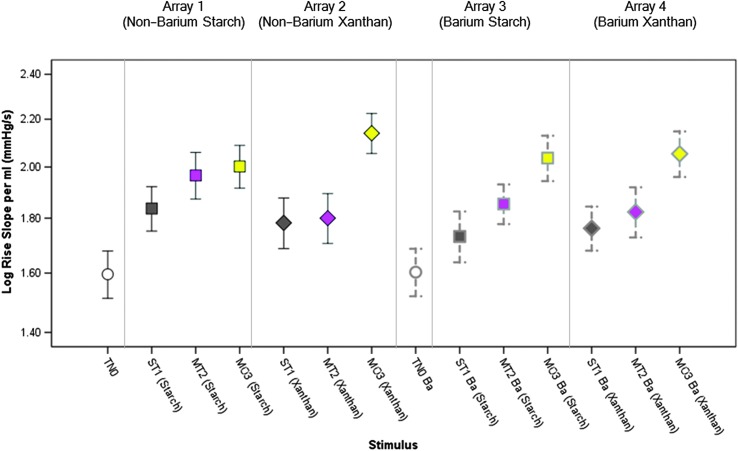
Means and 95% confidence interval boundaries for pressure rise slope by liquid flow level (within array). An overall pattern of increased pressure rise slope (*p* < .017) was seen for thicker liquids. Details regarding pairwise comparisons can be found in Table 2. TN0 = thin; ST1 = slightly thick; MT2 = mildly thick; MO3 = moderately thick; Ba = barium.

**Figure 5.**
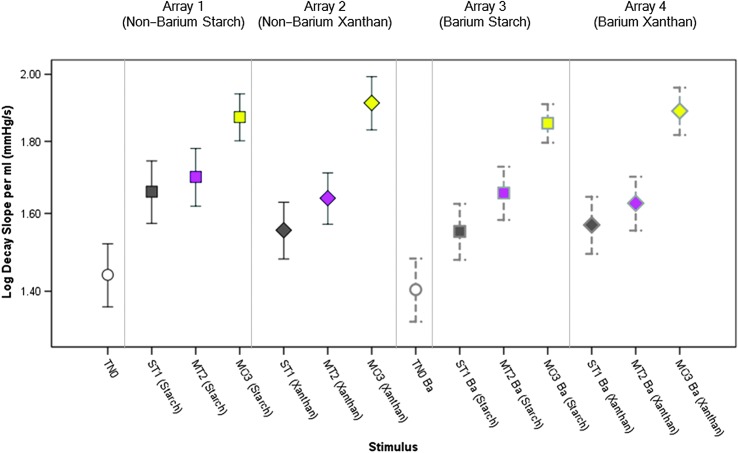
Means and 95% confidence interval boundaries for pressure decay slope by liquid flow level (within array). An overall pattern of increased pressure decay slope (*p* < .017) was seen for thicker liquids. Details regarding pairwise comparisons can be found in Table 2. TN0 = thin; ST1 = slightly thick; MT2 = mildly thick; MO3 = moderately thick; Ba = barium.

**Table 2.** Summary of results for the within-array repeated-measures analyses of variance (ANOVAs).

Parameter	Array	ANOVA result	Pairwise comparisons	Cohen's *d*
Log peak tongue pressure per ml (mmHg)	1 (Non–barium starch)	*F*(3, 400.08) = 13.25, *p* < .001	Level 0 < (1, 2, & 3); Level (1 & 2) < 3	0.33 (small) to 0.71 (medium)
	2 (Non–barium xanthan)	*F*(3, 404.08) = 11.24, *p* < .001	Level 0 < 1 & 3; Level 2 < 3	0.39 (small) to 0.77 (medium)
	3 (Barium starch)	*F*(3, 400.15) = 9.46, *p* < .001	Levels 0, 1, & 2 < 3	0.55 (small) to 0.99 (large)
	4 (Barium xanthan)	*F*(3, 404.111) = 10.14, *p* < .001	Levels 0, 1, & 2 < 3	0.59 (small) to 0.96 (large)
Log rise slope per ml (mmHg/s)	1 (Non–barium starch)	*F*(3, 342.12) = 5.67, *p* = .001	Level 0 < 1, 2, & 3	2.0 (large)
	2 (Non–barium xanthan)	*F*(3, 337.01) = 5.66, *p* = .001	Level 2 < 3	0.52 (medium)
	3 (Barium starch)	*F*(3, 352.68) = 3.64, *p* = .013	Levels 0, 1, & 2 < 3	0.41 (small) to 1.0 (large)
	4 (Barium xanthan)	n.s.	Trend of higher slopes with thicker consistencies	
Log decay slope per ml (mmHg/s)	1 (Non–barium starch)	n.s.	Trend of higher slopes with thicker consistencies	
	2 (Non–barium xanthan)	*F*(3, 337.57) = 5.76, *p* = .001	Level 2 < 3	0.63 (medium)
	3 (Barium starch)	n.s.	Trend of higher slopes with thicker consistencies	
	4 (Barium xanthan)	*F*(3, 341.82) = 5.45, *p* = .001	Level 2 < 3	0.45 (small)

*Note.* n.s. = not significnant.

### Variations in Tongue Pressure Measures Across Gravity Flow Matched Liquids

**Figure 6.**
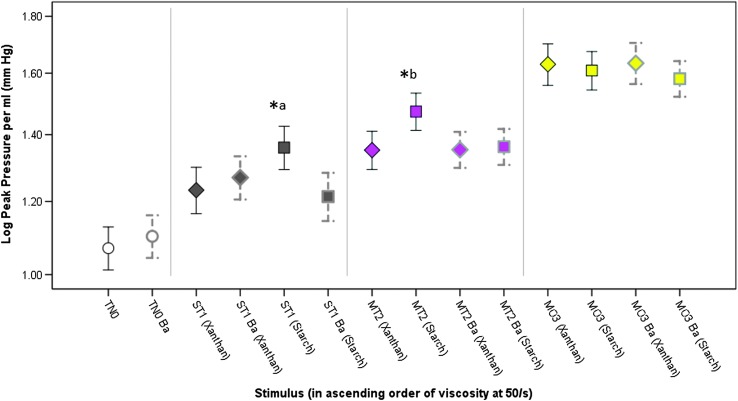
Means and 95% confidence interval boundaries for peak pressure amplitude by liquid flow level (across arrays). When peak pressures were compared across the different stimulus arrays within IDDSI flow levels, few differences were seen across stimuli that were closely matched for gravity flow. In the slightly thick (*a) and mildly thick (*b) categories, significantly higher (*p* < .17) peak pressure amplitudes were seen for the nonbarium, starch-thickened stimuli. TN0 = thin; ST1 = slightly thick; MT2 = mildly thick; MO3 = moderately thick; Ba = barium.

Figure 6 illustrates the differences seen in peak tongue pressure across the different liquids (nonbarium vs. barium; starch vs. xanthan gum thickeners) within IDDSI Levels 0–3. The stimuli are arranged in order of ascending viscosity at 50/s (as summarized in Table 1). It can be seen that peak pressures cluster quite closely across the liquids within each IDDSI category (noting that gravity flow was matched to within a 1-ml range, which is smaller than the range spanned by each IDDSI category). What is most obvious, however, is that the stimuli eliciting the highest peak pressures were not the liquids with the highest viscosity and there was no obvious pattern of differences related to the inclusion of barium or between thickening agents. Rather, significantly higher peak pressures (but with small effect sizes) were seen with the starch-thickened nonbarium stimuli in the slightly, *F*(3, 411.03) = 17.11, *p* < .001*, d* = 0.25, and mildly thick categories, *F*(3, 397.23) = 16.28, *p* < .001*, d* = 0.38. Similar results were seen for comparisons of rise slope per milliliter across the gravity flow–matched stimuli. Significantly higher rise slopes were seen for the nonbarium, starch-thickened stimulus compared with the two slightly thick barium stimuli, *F*(3, 353.27) = 5.41, *p* = .001, *d* = 0.29, that is, small. For the mildly thick liquids, the nonbarium, starch-thickened stimulus elicited a significantly higher rise slope compared with the other three stimuli, *F*(3, 345.24) = 8.19, *p* < .001*, d* = 0.26, that is, small.

With respect to comparisons of decay slope, a significantly higher decay slope was seen for the nonbarium, starch-thickened slightly thick stimulus compared with the other Level 1 IDDSI liquids, *F*(3, 353.48) = 8.20, *p* < .001, *d* = 0.32, that is, small. However, in the mildly thick category, the highest decay slope was seen with the xanthan gum–thickened barium stimulus, *F*(3, 345.28) = 4.29, *p* = .005, *d* = 0.29, that is, small.

## Discussion

The results of this study confirm that healthy young adults modulate sip volume and the amplitudes and slopes of tongue–palate pressure when swallowing liquids of different consistencies. The sip volumes found in this study are consistent with those reported in previous studies of natural discrete swallows of water (Bennett, van Lieshout, Pelletier, & Steele, 2009). Findings of smaller sips with thicker consistencies are also consistent with the literature (Steele & van Lieshout, 2005). In contrast to previous research exploring sip size with barium (Steele & van Lieshout, 2005), we did not observe significantly smaller sips with barium compared with nonbarium stimuli; however, the barium concentration in this study was dramatically lower (i.e., 20% w/v) than the 250% w/v concentration used by Steele and van Lieshout (2005). Barium concentrations of 20%–40% w/v are currently understood to be optimal for videofluoroscopic examinations of oropharyngeal swallowing, to ensure sufficient opacity on the image while limiting coating of the mucosa (Steele et al., 2013).

One possible confounding factor that was not controlled in this study is the fact that flavor suppression can occur with the use of thickening agents (Christensen, 1980). Consequently, we cannot rule out the possibility that the smaller sip sizes seen with the thicker liquids arose, in part, due to differences in taste and/or flavor.

A limitation of this study is the fact that we are unable to rule out the possibility that some of the participants performed more than one swallow per bolus, particularly with larger sip sizes. As mentioned in the Method section, all measurements were performed on a single (initial) swallow for each bolus. Preliminary analysis of videofluoroscopic data collected in a separate data collection session from the same participants, using the same barium liquids and same instructions to take comfortable single sips from a cup containing 40 ml, shows that both the mean and mode scores for number of swallows per bolus were 1, suggesting that clearing swallows were not common in these participants (Steele et al., 2018).

The findings of stronger pressures and steeper slopes (indicating greater magnitudes of pressure rise or decay per unit of time) with thicker consistencies confirmed our hypotheses and are clearly seen when tongue pressure parameters are expressed as units per milliliter of bolus, controlling for the influence of sip volume variations that occur both as a factor of liquid thickness (i.e., smaller sip sizes for thicker consistencies) and of administration method (i.e., smaller sip volumes for liquids administered by teaspoon compared with those sipped from a cup). Evidence of significantly higher pressures and more rapid pressure decay with thicker liquids is consistent with previous evidence and the idea that liquids with higher viscosity require higher forces for flow initiation and, conversely, that thinner liquids might elicit a longer period of active bolus control by the tongue, reflected in the form of more gradual pressure decay (Steele, Bailey, & Molfenter, 2010).

It had been our original intent to include IDDSI Level 4 extremely thick liquids in this study, to study the full range of thickened liquids used in clinical practice. Extremely thick liquids achieve a ceiling effect on the IDDSI Flow Test, and our original assumption was that this similar saturated result across starch- and gum-thickened extremely thick liquids would justify grouping them in a single flow level and comparing them. However, inspection of other rheological characteristics of these stimuli showed a very wide range in viscosity between the starch- and gum-thickened extremely thick liquids, suggesting that, although they test similarly on the IDDSI Flow Test (Cichero et al., 2017; Hanson, 2016), they are not comparable. Future studies will definitely be needed to dig into the question of whether tongue pressures or swallowing physiology differ across liquids above the upper boundary for moderately thick liquids according to the IDDSI framework (Cichero et al., 2017).

Importantly, this study confirmed the absence of significant differences in tongue pressures between thin barium and thin nonbarium stimuli. This result is exciting because it supports the widely held clinical assumption that thin barium stimuli elicit similar swallowing behaviors to thin nonbarium stimuli such as water, at least with respect to tongue pressures. With the thicker stimuli, this finding also held true. Thus, the study provides general support for the common practice of generalizing findings seen with barium stimuli in videofluoroscopy to nonbarium stimuli with similar flow characteristics outside the assessment context. Of course, it must be emphasized that the barium stimuli in this study were prepared at a low concentration of 20% w/v and that assumptions of similarity with nonbarium stimuli may not hold with higher barium concentrations (Dantas et al., 1989; Stokely et al., 2014).

The study results also bring clarity regarding the differences that can be expected between liquids thickened with different thickening agents. As a rule, we found no pattern of differences in tongue pressure related to the use of starch- versus xanthan gum-based thickening agents, provided the stimuli were tightly matched with respect to gravity flow. Figures 3 and 6 illustrate an interesting result with significantly higher peak pressure amplitudes for the slightly and mildly thick starch-thickened nonbarium stimuli (Array 1) compared with the other thickener–barium combinations. Why the use of a starch thickener without barium might lead to these higher pressure amplitudes and higher pressure slopes is not clear. These stimuli did not have the highest viscosity or the highest density within either the slightly or mildly thick levels. It is possible that taste, mouthfeel (Ong et al., 2018), or other rheological properties such as yield stress contributed to the observed patterns, but further research would be needed to explore these possibilities.

A limitation of the data in this study is the fact that they were collected without visualization of the bolus under videofluoroscopy. As such, it is not yet known how the observed modulations of tongue pressure influence bolus flow through the oropharynx, and a future study to answer this question is definitely needed.

## Conclusions

Overall, the findings of this study lend strong support to the idea that differences in consistency, measured using the IDDSI Flow Test (Cichero et al., 2017; Hanson, 2016) elicit variations in tongue pressure during swallowing. Future videofluoroscopic studies will be needed to further elucidate the nature and magnitude of differences in swallowing physiology and bolus flow that occur across the IDDSI levels, while controlling for the sip volume and tongue pressure variations documented in this study.

## Supplementary Material

10.1044/2018_JSLHR-S-18-0229SMS1 Supplemental Material S1. Descriptive statistics: tongue pressure parameters during swallows of barium and non-barium liquids with different consistencies.
