# Supplementary material for: Modulation of Tongue Pressure According to Liquid Flow Properties in Healthy Swallowing
Source: J Speech Lang Hear Res. 2019 Jan 25;62(1):22–33. doi: 10.1044/2018_JSLHR-S-18-0229 (PMC6437699; doi:10.1044/2018_JSLHR-S-18-0229)
Supplement: Supplemental Material S1. [file JSLHR-62-22-s001.pdf]

Supplemental Material S1. Descriptive statistics: tongue pressure parameters during swallows of barium and non-barium liquids with different consistencies.

These descriptive statistics reflect means and 95% confidence intervals for tongue-palate pressure peak amplitude (in mmHg), pressure rise slope (in mm Hg/s) and pressure decay slope (in mm Hg/s). The statistical analyses were performed using log-transformed tongue pressure parameters to correct for non-normalized distribution of residuals. Both the transformed and non-transformed values are provided here, for reference. Additional details regarding the methods of data collection and signal processing can be found in the main article.

It is important to recognize that a confounding effect of variations in sip volume was found in the data analysis, with significantly smaller sips for thicker consistencies. Consequently, the supplementary data are presented as follows:
1) Descriptive statistics for sip volume (in ml) by stimulus;
2) Tongue pressure parameter data by stimulus, in units of <<tongue-pressure parameter per ml>>;
3) Tongue pressure parameter data by stimulus, *per bolus*, without translation to units of <<tongue-pressure parameter per ml>>.

Sip Volume (ml)

Stimulus	Mean	95% Confidence Interval Lower Bound	95% Confidence Interval Upper Bound
Thin Non-Barium	9.89	8.65	11.12
Slightly thick Non-Barium (Starch)	6.28	5.38	7.17
Slightly thick Non-Barium (Xanthan)	7.87	6.81	8.93
Mildly thick Non-Barium (Starch)	5.14	4.63	5.65
Mildly thick Non-Barium (Xanthan)	6.31	5.61	7.01
Moderately thick Non-Barium (Starch)	4.12	3.71	4.53
Moderately thick Non-Barium (Xanthan)	3.90	3.31	4.48
Thin Barium	9.61	8.52	10.70
Slightly thick Barium (Starch)	7.66	6.69	8.62
Slightly thick Barium (Xanthan)	7.69	6.65	8.74
Mildly thick Barium (Starch)	6.11	5.54	6.68
Mildly thick Barium (Xanthan)	6.45	5.80	7.10
Moderately thick Barium (Starch)	4.30	3.84	4.77
Moderately thick Barium (Xanthan)	3.79	3.26	4.31

Descriptive statistics for the tongue pressure parameters (in units of <<pressure per ml>>), both in regular units and in log-transformed units, as described in the manuscript:

	Peak Pressure per ml (mmHg)			Log Peak Pressure per ml (mmHg)		
**Stimulus**	*Mean*	*95% Confidence Interval Lower Bound*	*95% Confidence Interval Upper Bound*	*Mean*	*95% Confidence Interval Lower Bound*	*95% Confidence Interval Upper Bound*
Thin Non-Barium	14.84	12.97	16.70	1.09	1.03	1.15
Slightly thick Non-Barium (Starch)	29.47	24.79	34.16	1.35	1.28	1.42
Slightly thick Non-Barium (Xanthan)	24.74	20.46	29.02	1.27	1.21	1.34
Mildly thick Non-Barium (Starch)	40.57	31.15	49.98	1.48	1.41	1.54
Mildly thick Non-Barium (Xanthan)	29.47	24.98	33.96	1.36	1.30	1.43
Moderately thick Non-Barium (Starch)	56.53	45.66	67.41	1.62	1.55	1.69
Moderately thick Non-Barium (Xanthan)	67.08	54.20	79.96	1.68	1.61	1.76
Thin Barium	16.53	14.32	18.75	1.12	1.05	1.18
Slightly thick Barium (Starch)	22.31	19.36	25.27	1.24	1.18	1.31
Slightly thick Barium (Xanthan)	25.51	21.93	29.10	1.30	1.23	1.37
Mildly thick Barium (Starch)	28.62	25.00	32.24	1.38	1.32	1.43
Mildly thick Barium (Xanthan)	28.61	25.14	32.08	1.37	1.32	1.43
Moderately thick Barium (Starch)	51.89	43.06	60.72	1.61	1.54	1.67
Moderately thick Barium (Xanthan)	62.77	53.02	72.51	1.68	1.60	1.75

	Rise Slope per ml (mmHg/s)			Log Rise Slope per ml (mmHg/s)		
**Stimulus**	*Mean*	*95% Confidence Interval Lower Bound*	*95% Confidence Interval Upper Bound*	*Mean*	*95% Confidence Interval Lower Bound*	*95% Confidence Interval Upper Bound*
Thin Non-Barium	56.78	46.18	67.38	1.60	1.51	1.68
Slightly thick Non-Barium (Starch)	102.17	82.96	121.38	1.84	1.75	1.92
Slightly thick Non-Barium (Xanthan)	118.08	76.95	159.21	1.78	1.69	1.88
Mildly thick Non-Barium (Starch)	170.15	116.54	223.75	1.97	1.87	2.06
Mildly thick Non-Barium (Xanthan)	109.37	69.85	148.89	1.80	1.71	1.89
Moderately thick Non-Barium (Starch)	177.48	118.99	235.98	2.00	1.92	2.09
Moderately thick Non-Barium (Xanthan)	209.66	163.24	256.08	2.14	2.06	2.22
Thin Barium	59.86	48.69	71.04	1.60	1.52	1.69
Slightly thick Barium (Starch)	103.65	68.39	138.91	1.73	1.64	1.83
Slightly thick Barium (Xanthan)	96.43	65.34	127.52	1.76	1.68	1.84
Mildly thick Barium (Starch)	106.02	80.28	131.76	1.85	1.78	1.93
Mildly thick Barium (Xanthan)	128.11	85.67	170.55	1.82	1.73	1.92
Moderately thick Barium (Starch)	197.80	141.42	254.18	2.04	1.94	2.13
Moderately thick Barium (Xanthan)	201.46	140.68	262.25	2.05	1.96	2.15

	Decay Slope per ml (mmHg/s)			Log Decay Slope per ml (mmHg/s)		
**Stimulus**	*Mean*	*95% Confidence Interval Lower Bound*	*95% Confidence Interval Upper Bound*	*Mean*	*95% Confidence Interval Lower Bound*	*95% Confidence Interval Upper Bound*
Thin Non-Barium	39.72	31.62	47.82	1.44	1.36	1.52
Slightly thick Non-Barium (Starch)	68.54	55.62	81.46	1.66	1.57	1.74
Slightly thick Non-Barium (Xanthan)	50.23	41.68	58.79	1.56	1.48	1.63
Mildly thick Non-Barium (Starch)	77.91	58.15	97.66	1.70	1.62	1.78
Mildly thick Non-Barium (Xanthan)	57.65	48.53	66.76	1.64	1.57	1.71
Moderately thick Non-Barium (Starch)	98.96	83.04	114.88	1.87	1.80	1.94
Moderately thick Non-Barium (Xanthan)	116.93	92.98	140.88	1.91	1.83	1.99
Thin Barium	36.19	29.59	42.79	1.40	1.33	1.48
Slightly thick Barium (Starch)	48.52	41.59	55.45	1.55	1.48	1.63
Slightly thick Barium (Xanthan)	51.13	41.14	61.13	1.57	1.49	1.64
Mildly thick Barium (Starch)	64.34	50.62	78.06	1.66	1.58	1.73
Mildly thick Barium (Xanthan)	56.97	48.47	65.47	1.63	1.55	1.70
Moderately thick Barium (Starch)	86.32	75.43	97.21	1.85	1.80	1.91
Moderately thick Barium (Xanthan)	104.70	84.59	124.81	1.89	1.82	1.96

Descriptive statistics for the tongue pressure parameters *per bolus*, without translation to <<units of pressure per ml>>:

Peak Pressure (mmHg)

**Stimulus**	*Mean*	*95% Confidence Interval Lower Bound*	*95% Confidence Interval Upper Bound*
Thin Non-Barium	126.22	110.89	141.54
Slightly thick Non-Barium (Starch)	143.83	127.10	160.56
Slightly thick Non-Barium (Xanthan)	143.00	127.68	158.32
Mildly thick Non-Barium (Starch)	159.79	143.82	175.75
Mildly thick Non-Barium (Xanthan)	144.40	129.20	159.60
Moderately thick Non-Barium (Starch)	181.77	163.62	199.93
Moderately thick Non-Barium (Xanthan)	175.30	157.15	193.45
Thin Barium	136.97	119.65	154.29
Slightly thick Barium (Starch)	138.60	123.25	153.94
Slightly thick Barium (Xanthan)	151.18	133.87	168.49
Mildly thick Barium (Starch)	149.39	135.89	162.89
Mildly thick Barium (Xanthan)	154.55	139.25	169.84
Moderately thick Barium (Starch)	170.71	154.51	186.91
Moderately thick Barium (Xanthan)	169.64	152.20	187.08

	Rise Slope per ml (mmHg/s)		
**Stimulus**	*Mean*	*95% Confidence Interval Lower Bound*	*95% Confidence Interval Upper Bound*
Thin Non-Barium	471.46	390.94	551.97
Slightly thick Non-Barium (Starch)	521.46	423.77	619.15
Slightly thick Non-Barium (Xanthan)	597.40	467.23	727.58
Mildly thick Non-Barium (Starch)	653.37	513.73	793.02
Mildly thick Non-Barium (Xanthan)	484.79	384.24	585.34
Moderately thick Non-Barium (Starch)	522.90	428.69	617.11
Moderately thick Non-Barium (Xanthan)	571.02	493.47	648.57
Thin Barium	483.40	405.93	560.88
Slightly thick Barium (Starch)	552.74	440.91	664.57
Slightly thick Barium (Xanthan)	499.41	413.38	585.43
Mildly thick Barium (Starch)	547.94	447.88	648.00
Mildly thick Barium (Xanthan)	584.19	457.01	711.37
Moderately thick Barium (Starch)	617.59	484.89	750.29
Moderately thick Barium (Xanthan)	543.96	405.14	682.71

**Stimulus**	**Decay Slope per ml (mmHg/s)** *Mean*	*95% Confidence Interval Lower Bound*	*95% Confidence Interval Upper Bound*
Thin Non-Barium	323.01	268.70	377.33
Slightly thick Non-Barium (Starch)	326.53	276.45	376.60
Slightly thick Non-Barium (Xanthan)	297.11	256.52	337.70
Mildly thick Non-Barium (Starch)	314.59	263.18	366.00
Mildly thick Non-Barium (Xanthan)	297.43	255.71	339.15
Moderately thick Non-Barium (Starch)	359.77	302.67	416.87
Moderately thick Non-Barium (Xanthan)	334.83	285.73	383.92
Thin Barium	290.67	245.04	336.29
Slightly thick Barium (Starch)	308.04	263.83	352.24
Slightly thick Barium (Xanthan)	306.92	262.06	351.78
Mildly thick Barium (Starch)	325.08	278.29	371.87
Mildly thick Barium (Xanthan)	316.38	268.17	364.58
Moderately thick Barium (Starch)	321.07	279.84	362.30
Moderately thick Barium (Xanthan)	302.86	257.13	348.58
